# Novel mutations in *CRYGD* are associated with congenital cataracts in Chinese families

**DOI:** 10.1038/srep18912

**Published:** 2016-01-06

**Authors:** Guoxing Yang, Zhimin Chen, Wulin Zhang, Zhiqiang Liu, Jialiang Zhao

**Affiliations:** 1 Department of Opthalmology, Peking Union Medical College Hospital, Chinese Academy of Medical Sciences & Peking Union Medical College, Beijing, China; 2 Department of Opthalmology, Hebei Provincial Ophthalmic Hospital, Hebei, China; 3 Hebei Provincial Key laboratory of ophthalmology, Hebei, China

## Abstract

Congenital cataract disease is a clinically and genetically heterogeneous lens disorder. The purpose of this study was to identify the genetic defects and to investigate the relationships between disease-causing genes and lens morphology in congenital cataracts. Patients were given a physical examination, and their blood samples were collected for DNA extraction. Mutation analysis was performed by direct sequencing of the following candidate genes: *CRYGC, CRYGD*, *CRYGS, GJA8, GJA3* and *CRYAA*. Mutational analysis of *CRYGD* identified a recurrent (p.P24T) mutation in two unrelated families with congenital coralliform cataracts and three novel (p.Q101X, p.E104fsX4 and p.E135X) mutations in three families with congenital nuclear cataracts. The p.E135X mutation is a *de novo* mutation. Haplotype analysis showed patients inherited the same *CRYGD* allele originated from father. The p.E135X mutation seen in two siblings suggests a mechanism of gonadal mosaicism in the father.

Congenital cataracts are common and cause approximately one third of infant blindness occurring in approximately 1–6 of every 10,000 live births. One quarter of congenital cataract disease cases are hereditary^1^,^2^,^3^,^4^.

Congenital cataract disease is a clinically and genetically heterogeneous lens disorder. Cataracts that are phenotypically identical can result from mutations at different genetic loci and can have different inheritance patterns. Conversely, cataracts with dissimilar phenotypes may result from mutations in a single gene or gene family. It is believed that the type of genetic mutation is related to the morphology of the cataract^5^. To date, about 40 genetic loci have been linked to congenital cataracts, and 26 genes have been cloned and sequenced, including crystallins, connexins, heat shock transcription factor-4, aquaporin-0 and beaded filament structural protein-2^5^.

In this study, we collected information on five families with congenital cataracts. In the follow-up genetic study, we identified four mutations in the *CRYGD* that is responsible for the disease.

## Results

### Clinical findings

Patients suffering from bilateral cataracts were diagnosed at childhood in all five families. Two families afflicted with congenital coralliform cataracts were enrolled in the Jiangxi province and the Hebei province respectively in this study. Three families afflicted with congenital nuclear cataracts had completed cataract operation in all patients. None of the participants had any other related ophthalmic syndromes.

### Mutation analysis

**Figure 1:**
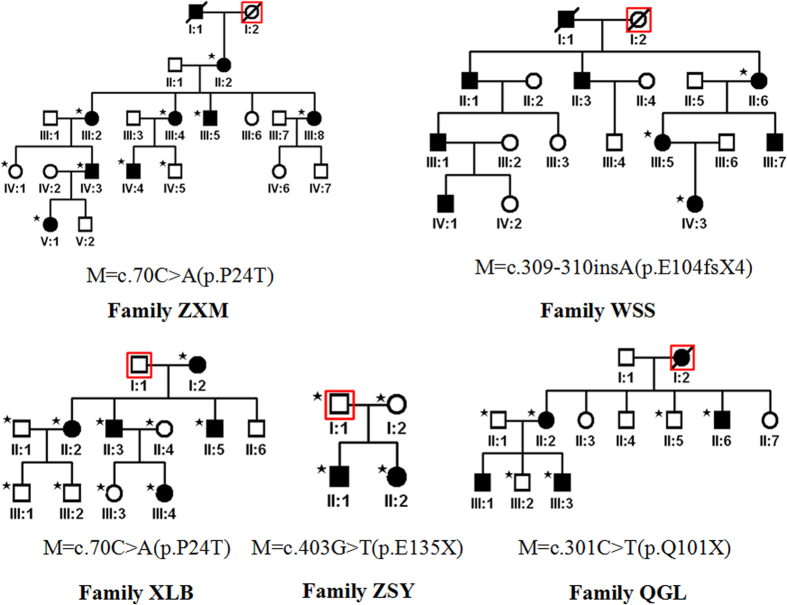
The pedigrees of five families with congenital cataract (Asterisk is represented the participant).

**Figure 2:**
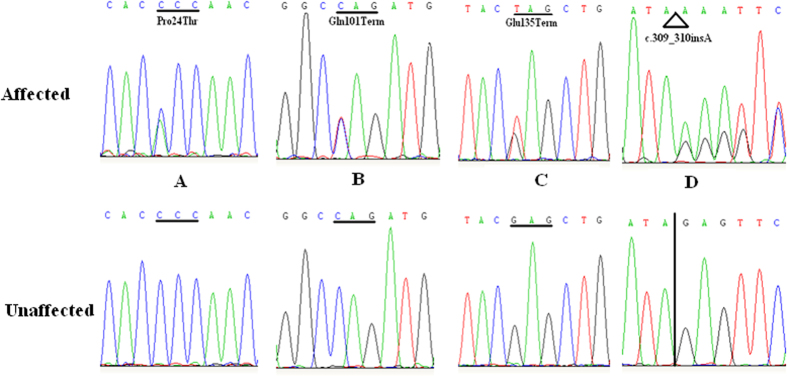
DNA sequences of *GRYGD* in affected and control individuals. Four heterozygous changes c.70C > A, c.301C > T, c.309_310insA and c.403G > T were found in the affected individuals.

By directly sequencing the coding region of *CRYGD*, we identified a recurrent (p.P24T) mutation in two unrelated families with congenital coralliform cataracts and three novel (p.Q101X, p.E104fsX4 and p.E135X) mutations in three families with congenital nuclear cataracts (Figs 1 and 2). The p.E135X mutation was identified in two affected individuals but was not found in their parents in family ZSY. These mutations were not found in healthy relatives or the 100 control populations from the same ethnic background.

### Paternity testing and haplotype analysis

**Figure 3:**
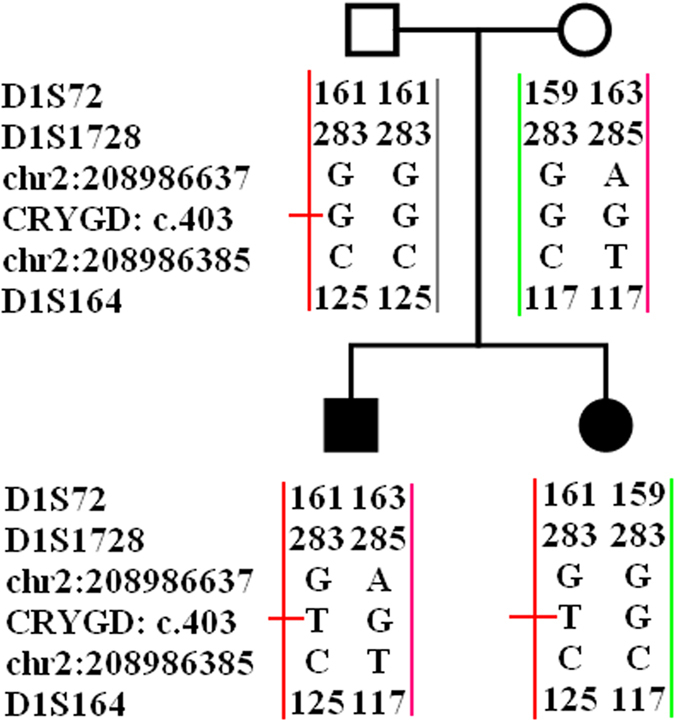
Paternity testing and haplotype analysis in family ZSY. Two patients inherited the same *CRYGD* allele from their father.

Paternity testing confirmed that the parents of the patients are biological parents in family ZSY. Haplotype analysis showed that two patients inherited the same *CRYGD* allele from their father (Fig. 3).

## Discussion

Twenty mutations in *CRYGD* have previously been reported (three in our study) (Table 1)^6^,^7^,^8^,^9^,^10^,^11^,^12^,^13^,^14^,^15^,^16^,^17^,^18^,^19^,^20^,^21^,^22^. In the present study, we identified three novel mutations in CRYGD, which are predicted to cause premature stop codon and lead to the deletion of the C-terminal of protein.

CRYGD composes of two similar domains, in which each domain composes of two similar motifs. Each motif, about forty amino acid residues long, is folded in a distinctive ‘Greek key’ pattern. The missing residues at the C-terminus influence the folding of the Greek key motif (GKM) and then affect the biological functions of the protein.

Coralliform cataracts are a special form of congenital cataracts. Several studies have shown that mutations in the *CRYGD* gene can result in congenital coralliform cataracts, and the P24T mutation of *CRYGD* has been reported in multiple cases^8^. In the two autosomal dominant congenital coralliform cataract pedigrees in this study, we identified a recurrent P24T mutation. According to the reported pedigrees, most of the congenital coralliform cataracts resulted from *CRYGD* mutations. This information indicates that the coralliform phenotype and the *CRYGD* gene are closely related. Our results support the idea that virulence genes and lens morphology are related^23^,^24^,^25^,^26^,^27^,^28^,^29^. Results of biophysical analysis have shown that the P24T mutant protein has a significantly lower solubility than wild-type human γD crystalline^30^,^31^.

The p.E135X mutation in family ZSY was found in two patients, but the mutation was not observed in the parents of either patient. The haplotype analysis showed that the father shares the common chromosome fragment with the children, indicating that the fathers are in a state of gonadal mosaicism^32^.

## Methods

### Patients and clinical data

Families enrolled in this study were from the Inner Mongolia Autonomous Region, Jiangxi province, Hebei province and Shandong province of China. All patients had undergone cataract surgery. Clinical examination, peripheral blood collection and DNA extraction were performed in the Department of Ophthalmology at the Peking Union Medical College Hospital. This study followed the tenets of the Declaration of Helsinki and was approved by the Ethics Committee of the Peking Union Medical College Hospital. The methods were carried out in accordance with the approved guidelines. Written informed consent was obtained from all participants. Clinical data for these subjects was collected through detailed ocular examinations. In addition, physical examinations were performed to exclude systemic diseases.

### Mutation analysis

*CRYGC, CRYGD*, *CRYGS, GJA8, GJA3* and *CRYAA* were selected as candidate genes. All coding exons for these genes were amplified by polymerase chain reaction (PCR) using a set of previously described paired primers. The PCR products were sequenced using an ABI3730 Automated Sequencer (PE Biosystems, Foster City, CA, USA).

### Paternity testing and haplotype analysis

Golden*e*ye^TM^20A was used to mark up the allele in different chromosomes in members of the family ZSY. Markers surrounding the gene and inside the gene were analysed to determine the haplotype of the ZSY family.

## Additional Information

**How to cite this article**: Yang, G. *et al.* Novel mutations in *CRYGD* are associated with congenital cataracts in Chinese families. *Sci. Rep.* **6**, 18912; doi: 10.1038/srep18912 (2016).

## Figures and Tables

**Table 1:** Human *CRYGD* mutations associated with childhood cataracts.

Exon	Nucleotide	Amino acid	Inheritance	Phenotype	Origin	Ref.
Ex2	c.43C > T	p.R15C	AD	Punctate, Coralliform	US, China	6
Ex2	c.43C > A	p.R15S	AD	Coraliform	China	7
Ex2	c.70C > A	p.P24T	AD	Coraliform, lamellar, fasciculiform, Acueliform	India, China, Morocco, Saudi Arabia, Australia, Europe	8
Ex2	c.70C > T	p.P24S	AD	Polymorphic	Mid-Asia	9
Ex2	c.106G > C	p.A36P	AD	Nuclear	China	10
Ex2	c.109C > A	p.R37S	S	crystals	China, Czech Rep	11
Ex2	c.110G > C	p.R37P	AD	Nuclear	China	12
Ex2	c.127T > C	p.W43R	AD	Nuclear	China	13
Ex2	c.168C > G	p.Y56X	AD	Nuclear	Brazil	14
Ex2	c.176G > A	p.R59H	AD	Aculeiform	Switzerland,Mexico	15
Ex2	c.181G > C	p.G61C	AD	Coralliform	China	16
Ex2	c.229C > A	p.R77S	AD	Anterior polar coronary	India	17
Ex3	c.301C > T	p.Q101X	AD	Nuclear	China	In this study
Ex3	c.309_310insA	p.E104fsX4	AD	Nuclear	China	In this study
Ex3	c.320A > C	p.E107A	AD	Nuclear	Mexico	18
Ex3	c.402C > A	p.Y134X	AD	Microcornea	Denmark	19
Ex3	c.403G > T	p.E135X	AD	Nuclear	China	In this study
Ex3	c.418C > T	p.R140X	AD	Nuclear	India, Ashkenazi Jewish	20
Ex3	c.470G > A	p.W157X	AD	Central nuclear	India	21
Ex3	c.494delG	p.G165AfsX3	AD	Nuclear	China	22
